# Types and drug susceptibility patterns of bacterial isolates from eye discharge samples at Gondar University Hospital, Northwest Ethiopia

**DOI:** 10.1186/1756-0500-7-292

**Published:** 2014-05-12

**Authors:** Dagnachew Muluye, Yitayih Wondimeneh, Feleke Moges, Tesfaye Nega, Getachew Ferede

**Affiliations:** 1 School of Biomedical and Laboratory Sciences, College of Medicine and Health Sciences, University of Gondar, P.O. Box 196, Gondar, Ethiopia; 2 Unit of Bacteriology, Gondar University Hospital, P.O. Box 196, Gondar, Ethiopia

**Keywords:** Bacteria isolates, Drug susceptibility pattern, Eye discharge

## Abstract

**Background:**

The type and pattern of organisms that cause ocular infection changes over time. Moreover, the causative organisms have developed increased drug resistance. Therefore, the aim of this study was to determine the prevalent bacterial agents of eye discharge and their drug susceptibility patterns to commonly used antimicrobial agents.

**Methods:**

A retrospective study was conducted at Gondar University Hospital, Northwest Ethiopia from September, 2009 to August, 2012. Culture and drug susceptibility test results of patients who had eye infections were taken for analysis. Eye discharge samples were cultured on MacConkey agar, blood agar and chocolate agar plates. A standard biochemical procedure was used for full identification of bacterial isolates. Antimicrobial susceptibility tests were done on Mueller-Hinton agar by using disk diffusion method. Data was entered and analyzed by using SPSS version 16 software.

**Result:**

A total of 102 eye discharges were submitted for microbiological evaluation, of which (60.8%) had bacterial growth. The most frequently isolated bacterial isolates were gram-positive bacteria (74.2%). The predominant bacterial species isolated was Coagulase-negative staphylococci (27.4%) followed by *S. aureus (*21%). Within the age group of 1 day-2 years old, (66.1%) of bacteria were isolated. Most of the bacterial isolates were resistance to ampicilin (71%), amoxicilin (62.9%), erythromycin (43.5%), gentamicin (45.2%), penicillin (71%), trimethoprim-sulphamethoxazole (58.1%), and tetracycline (64.6%) while Ceftriaxon and Ciprofloxacin showed (75.8%) and (80%) susceptibility respectively. From the total bacterial isolates, (87.1%) were showed multi drug resistance (MDR) to two or more drugs.

**Conclusion:**

The prevalence of bacterial isolates in eye discharge was high in the study area and majority of isolates were gram-positive bacteria. Most of the bacterial isolates were resistant to frequently used antimicrobials. Therefore, drug susceptibility test is necessary before prescribing any antimicrobials.

## Background

Eye is one of the sense organ which is important throughout our life. The awareness given to eye health and cleanliness is vital due to many factors. Dust, high temperature, microorganisms and other factors can lead to various eye diseases which can lead to blindness. The clinical signs and symptoms of inflammation of the eyes, in the presence of mucous pus are frequently caused by bacteria, the formation of pus increase, conjunctival hyperemia and lid edema [1,2].

Bacteria causes eye disease because of their virulence and host's condensed fighting from various factors such as socio-economic status, individual hygiene, lifestyle, nutrition, inheritance, physiology, and age [3]. Eye may be infected by being exposed to outside influences and internal invasion of bacteria that are transported by the blood stream [4]. External microbial infections of the eye are usually centralized in one place but may frequently distributed to other tissues. The conjunctiva and eyelid have a normal microbial flora controlled by its own mechanism and by the host. Any change of this normal flora leads to ocular infections [5,6].

Bacterial conjunctivitis is an inflammatory condition of the conjunctiva that results from infection due to one or more bacterial species. Most cases of acute bacterial conjunctivitis pointed eye are common and can affect both sexes and all age groups [7]. The common bacteria that causes eye infection are: *Pseudomonas aeruginosa, Proteus spp, Haemophilus aegyptius, Neisseria gonorrhoeae, Moraxella spp* such as *Moraxella catarrhalis, Moraxella lacunata, Streptococcus pyogenes,* and *Staphylococcus aureus*[8]*.* The microbial etiology and drug susceptibility as well as resistance profile may differ with geographic location according to the restricted inhabitants [9].

Bacterial eye infection needs instant institution of treatment. Treatment of bacterial eye infections may engross empirical treatment with topical ophthalmic broad-spectrum antibiotic formulations that become a prevailing practice among ophthalmologists and general practitioners. These jointly with irrational use of drugs, availability of antibiotics without prescription, have led to the development of resistance to commonly used antibiotics. Thus, the current trends in the etiology of bacteria that cause eye infections and their susceptibilities must be updated to make a rational choice of initial antibiotic therapy. The aim of this study was to determine bacterial isolates and drug susceptibility patterns of eye discharge at Gondar University Hospital.

## Methods

### Study design, area and period

A retrospective study was conducted at Gondar University Hospital, Northwest Ethiopia, from which procedures (collection of samples inoculation onto culture media, isolation and identification of bacterial strains, drug susceptibility testing) were carried out from September 2009 to August 2012. This University Hospital provides inpatient and outpatient services for more than 5 million inhabitants surrounding it.

### Study participants and data collection

The study participants were all patients’ who were clinically diagnosed with ocular infections and those who provide eye discharge sample at Gondar University Hospital during the study period. Socio-demographic and laboratory results which contain different bacterial isolates and drug susceptibility patterns of patients who had eye discharges were collected from the University Hospital Microbiology Laboratory unit registration books by using standard data collection format.

### Culture and identification

According to the standard operation procedures, eye discharge samples were collected by using sterile cotton swabs moisturized with normal saline solution and cultured on MacConkey agar, 5% Sheep's blood agar and chocolate agar plates. This was before the instillation of antimicrobial or steroidal eye drops for treatment. The isolation of bacteria was done by incubating the agar plates at temperature of 37°C for 24 and 48hs. Aerobic atmospheric condition was maintained for the MacConkey agar and blood agar, while 10% carbon dioxide (CO_2_) atmosphere was for the chocolate agar. Pure isolates of bacterial pathogen were preliminary characterized by colony morphology, gram-stain, and catalase test. A standard biochemical procedure was used for full identification of gram- positive and gram negative bacteria.

### Antimicrobial susceptibility testing

Antimicrobial susceptibility testing was performed for bacterial isolates by using agar diffusion method described by Bauer *et al*., 1966 on Mueller-Hinton agar (oxoide) [10]. The antimicrobial agents tested were: tetracycline (30 μg), erythromycin (15 μg), chloramphenicol (30 μg), gentamicin (10 μg), ciprofloxacin (5 μg), Trimethoprim-sulphamethoxazole (25 μg), ceftriaxone (30 μg), norflaxocin and amoxicillin (10 μg) (Oxoid, England). Resistance data were interpreted according to National Committee for Clinical Laboratory Standards (NCCLS). Reference strains of *E. coli* ATCC 25922**,***S. aureus* ATCC 25923 and *Pseudomonas aeruginosa* (ATCC 27853), were used for quality control for antimicrobial susceptibility tests [11].

### Statistical analysis

Statistical analysis was performed using SPSS version 16 software. The proportion of isolated bacteria with patient’s demographic information; and susceptibility to commonly used antibiotics was compared by using the Pearson Chi-square test. P-value ≤ 0.05 was considered as statistically significant.

### Ethical considerations

Ethical clearance was obtained from the Institutional Ethical Review Board of University of Gondar.

## Results

A total of 102 patients who gave eye discharge sample to bacteriological analysis were enrolled.

Of all, 65 (63.7%) were males and 37 (36.3%) were females. The mean age of the study subjects was 8.5 years, ranges from 1 day of life to 73 years old. Bacterial isolation in both sexes (P-value = 0.27) and various age groups (P-value = 0.59) was not showed statistically significant.

Out of 102 cultured eye discharges, 62 (60.8%) bacterial isolates were identified. The most frequently isolated bacterial isolates were gram-positive 46 (74.2%). The predominant bacterial species isolated was Coagulase-negative staphylococci (CONS) 17 (27.4%) followed by *S. aureus* 13 (21%) (Table 1).

**Table 1:** Bacteria isolated from samples of eye discharge at Gondar University Hospital (2009 to 2012)

**Type of bacteria**	**No. of isolates (%)**	**No. of isolates (%)**
CONS	17(27.4)	46(74.2)
*S. pneumoniae*	7(11.3)	
*S. pyogene*	9 (14.5)	
*S. aureus*	13(21)	
*E. coli*	5(8.1)	16(25.8)
NLF. gram neg. rods	2(3.2)	
*Klebsella spps*	9(14.5)	
Total	62(100)	

CONS = Coagulase negative Staphylococci.

NLF. gram neg. rods = non lactose fermented gram negative rode.

S. pneumoniae = Streptococcus pneumoniae.

S. pyogene = Streptococcus pyogene.

S. aureus = Staphylococcus aureus.

Within the age group of 1 day-2 years, 41 (66.1%) bacteria were isolated. Of these Coagulase-negative staphylococci accounts 11 (26.8%); and both S*. aureus and k. pneumoniae* accounts 7(17.1%) each (Table 2).

**Table 2:** Frequency of isolated bacteria in eye discharge in relation to sex and the various age groups at Gondar University Hospital (2009 to 2012)

**Isolated bacteria**	**Sex**		**Age distribution in years**				
	**Male**	**Female**	**≤ 2 yrs**	**3-11 yrs**	**12-17 yrs**	**18-39 yrs**	**≥40 yrs**
	**N (%)**	**N (%)**	**N (%)**	**N (%)**	**N (%)**	**N (%)**	**N (%)**
CONS	10(23.8)	7(35)	11(26.8)	2(50)	-	4(30.8)	-
*S. pneumoniae*	5(11.9)	2(10)	5(12.2)	-	1(33.3)	1(7.7)	-
*S. pyogene*	9(21.4)	-	5(12.2)	-	1(33.3)	2(15.4)	1(100)
*S. aureus*	10(23.8)	3(15)	7(17.1)	2(50)	1(33.3)	3(23.1)	-
*E. coli*	3(7.1)	2(10)	4(9.8)	-	-	1(7.7)	-
NLF. gram neg. rods	1(2.4)	1(5)	2(4.9)	-	-	-	-
*Klebsella spps*	4(9.5)	5(25)	7(17.1)	-	-	2(15.4)	-
**Total**	**42(67.7)**	**20(32.3)**	**41(66.1)**	**4(6.5)**	**3(4.8)**	**13(21)**	**1(1.6)**

N = number of isolates; CONS = Coagulase negative Staphylococci; % = percentage frequency.

NLF. gram neg. rods = non lactose fermented gram negative rode; − = Not isolated; yrs = years.

S. pneumoniae = Streptococcus pneumonia; S. pyogene = Streptococcus pyogene; S. aureus = Staphylococcus aureus.

Most of the bacterial isolates were resistant to ampicilin (71%), amoxicilin (62.9%), erythromycin (43.5%), gentamicin (45.2%), penicillin (71%), trimethoprim-sulphamethoxazole (58.1%), and tetracycline (64.6%). Ceftriaxon and Ciprofloxacin showed 75.8% and 80% susceptibility respectively (Table 3). The overall prevalence of multi drug resistance (MDR) to two or more drugs was observed in 54/62 (87.1%) of the isolated bacteria.

**Table 3:** Antimicrobial susceptibility patterns of isolated bacteria in eye discharge at Gondar University Hospital (2009 to 2012)

**Bacteria**	**No. of isolates**	**S/R**	**Antimicrobial agents**										
			**AMP**	**AMC**	**CRO**	**C**	**CIP**	**E**	**CN**	**NOR**	**PG**	**SXT**	**TTC**
			**No. (%)**	**No. (%)**	**No. (%)**	**No. (%)**	**No. (%)**	**No. (%)**	**No. (%)**	**No. (%)**	**No. (%)**	**No. (%)**	**No. (%)**
CONS	17	S	4(23.5)	6(35.3)	13(76.5)	8(47.1)	13(76.5)	6(35.3)	7(41.2)	10(58.8)	5(29.4)	5(29.4)	6(35.3)
		R	13(76.5)	11(64.7)	4(23.5)	9(52.9)	4(23.5)	11(64.7)	10(58.8)	7(41.2)	12(70.6)	12(70.6)	11(64.7)
*S. pneumoniae*	7	S	3(42.9)	2(28.6)	6(85)	5(71.4)	6(85.7)	6(85.7)	6(85.7)	4(57.1)	3(42.9)	4(57.1)	3(42.9)
		R	4(57.1)	5(71.4)	1(15)	2(28.6)	1(14.3)	1(14.3)	1(14.3)	3(42.9)	4(57.1)	3(42.9)	4(57.1)
*S. pyogene*	9	S	4(44.4)	4(44.4)	6(66.7)	6(66.7)	7(77.8)	7(77.8)	4(44.4)	6(66.7)	6(66.7)	5(55.6)	4(44.4)
		R	5(55.6)	5(55.6)	3(33.3)	3(33.3)	2(22.2)	2(22.2)	5(55.6)	3(33.3)	3(33.3)	4(44.4)	5(55.6)
*S. aureus*	13	S	3 (23.1)	6(46.2)	10(76.9)	9(69.2)	11(84.6)	7(53.8)	7(53.8)	8(61.5)	4(30.8)	4(30.8)	5(38.4)
		R	10(76.9)	7(53.8)	3(23.1)	4(30.8)	2(15.4)	6(46.2)	6(46.2)	5(38.5)	9(69.2)	9(69.2)	8(61.6)
*E. coli*	5	S	2(40)	1(20)	4(80)	3(60)	4(80)	-	4(80)	3(60)	-	2(40)	2(40)
		R	3(60)	4(80)	1(20)	2(40)	1(20)	5(100)	1(20)	2(40)	5(100)	3(60)	3(60)
NLF. gram neg. rods	2	S	-	1(50)	2(100)	1(50)	2(100)	-	1(50)	1(50)	-	2(100)	1(50)
		R	2(100)	1(50)	-	1(50)	-	2(100)	1(50)	1(50)	2(100)	-	1(50)
*Klebsella spps*	9	S	2(22.2)	3(33.3)	6(66.7)	6(66.7)	7(77.8)	-	5(55.6)	4(44.4)	-	4(44.4)	1(11.1)
		R	7(77.8)	6(66.7)	3(33.3)	3(33.3)	2(22.2)	9(100)	4(44.4)	5(55.6)	9(100)	5(55.6)	8(88.9)
**Total**	**62**	**S**	**18(29.0)**	**23(37.1)**	**47(75.8)**	**38(61.3)**	**50(80)**	**26(56.5)**	**34(54.8)**	**36(58)**	**18(29)**	**26(41.9)**	**22(35.4)**
		**R**	**44(71)**	**39(62.9)**	**15(24.2)**	**24(38.7)**	**12(20)**	**36(43.5)**	**28(45.2)**	**26(42)**	**44(71)**	**36(58.1)**	**40(64.6)**

CONS = Coagulase negative Staphylococci; S. pneumoniae = Streptococcus pneumoniae; S. pyogene = Streptococcus pyogene; S. aureus = Staphylococcus aureus; NLF. gram neg. rods = non lactose fermented gram negative rode; − = no sensitivity/resistance; AMP = Ampcillin; AMC = Amoxicillin-clavulanic acid; CRO = Ceftriaxone; CIP = Ciprofloxacin; C = Chloramphenicol; CN = Gentamicin; NOR = norfloxacilin; E = Erythromycin; P = Penicillin; SXT = Trimethoprim-sulphamethoxazole; TTC = Tetracycline.

## Discussion

In this study, the overall prevalence of bacterial eye infection was 60.8%. Similar findings have been reported in previous study conducted in Ethiopia, (54.2%) [12] and other countries such as: Niger (66.70%) [13], Nigeria, (69.2%) [14] and India (58.8%) [15]. The predominant bacterial isolates were Coagulase-negative staphylococci (27.42%) followed by *S. aureus* (20.97%). This finding is in agreement with previous study [12]. However, in other studies [15], the predominant isolates were *S. aureus* followed by *S. Pneumoniae.* This may be due to the difference in climate and geographical variations in different countries. Other isolates included *S. pneumoniae* (11.3%), *S. pyogene* (14.5%), *E. coli* (8.1%), *Klebsella spps* (14.5%), and non lactose fermentor gram negative rods (3.2%). These results are consistent with the study by Kasper *et al.,*[16]. Forty 40 (39.21%) samples were not showed bacterial growth. This might be due to the possibility of the presence of other micro-organisms which may cause eye infection such as viral causes or Chlamydia [17] or fungi causes especially yeasts [18].

The majority of the bacterial isolates, (66.1%) were from patients in the age range of less than two years of life. Susceptibility to infection is increased in babies because they are at a greater risk after their maternal immunity has been disappeared and before their own immunity system had matured [3]. In addition to this, the air plays an important role in the transfer of bacteria to hospital delivery rooms especially when opening the doors and windows which facilitates transfer it to the baby [19].

Commonly used antibiotics in a study area were; tetracycline, erythromycin, chloramphenicol, gentamicin, ciprofloxacin, Trimethoprim-sulphamethoxazole, penicillin, ceftriaxone, norflaxocin and amoxicillin. However, in the present study, different bacterial species had high level of resistance pattern to different antimicrobial agents. For example, Coagulase-negative staphylococci showed high level of resistance to ampicilin (76.5%), amoxicilin (64.7%), erythromycin and tetracycline each (64.7%), gentamicin (58.8%), penicillin and trimethoprim-sulphamethoxazole each (70.6%). This is in agreement with the previously studies [20]. The sensitivity of *Staphylococcus aureus* isolates to antimicrobials used showed the highest sensitivity to ciprofloxacin with percentage (84.6%) followed by ceftriaxone with percentage (76.9%) while the proportion was less sensitive to ampicilin with percentage(23.1%), penicillin and trimethoprim-sulphamethoxazole, (30.8%) each. This result is consistent with the previously studies [21]. It is well known fact that most *S. aureus* strains produce pencillinase and alternative penicillin binding proteins (PBP-2A) helps the organisms to become resistant to most beta lactam antibiotics [22].

In this study, most of bacterial isolates have shown high resistance to ampicilin (71%), penicillin (71%), amoxicilin (62.9%), tetracycline (64.6%), trimethoprim-sulphamethoxazole (58.1%), and erythromycin (43.5%). Similar findings have been reported in Iran [23] and in Aligarh [24]. Many studies reported indiscriminate use of antibiotics as the reason for drug resistance in microbial population [25] while ceftriaxon (75.8%) and ciprofloxacin (80%) showed susceptibility. This finding is comparable to other reports [26].

Prevalence of multidrug resistance (MDR) to two or more of bacterial isolates to the commonly prescribed antimicrobials was observed in 87.1% of the isolates. This is in agreement with the previous studies [27,28]. However, low prevalence of multidrug resistance was previously reported by Moreillon [22]. High prevalence of MDR in our study might be due to an irrational and unnecessary use of antimicrobial agents which can result in the emergence of bacterial strains that show multidrug resistance [29].

## Conclusion

The prevalence of bacterial isolates in eye discharge was high in the study area and majority of isolates were gram-positive bacteria. The predominant isolates were Coagulase-negative staphylococci and *S. aureus*. Most of the bacterial isolates were resistant to commonly used antimicrobials. Therefore, drug susceptibility test is essential before prescribing any antimicrobials.

### Limitation of the study

Due to the nature of the study, eye diagnosis is not clearly indicated and it is difficult to show whether the patients who underwent culture may have had chronic conjunctivitis and/keratitis and may have been treated earlier. Some of the bacterial isolates were reported as non-lactose fermenting gram negative rods and CN Staphylococci which are not specific. Moreover, there was no data about Chlamydia, Viral and other fungal eye infections.

## Competing interests

The authors declare that they have no competing interests.

## Authors’ contributions

DM: participated in conception and design of the study, data collection and analysis, interpretation of the findings, reviewed the manuscript. YW: Participated in conception and design of the study, data analysis and interpretations of the findings, reviewed the manuscript. FM: participated in conception and design of the study, interpretation of the findings, reviewed the manuscript. TN: Participated in conception and design of the study, data collection, reviewed the manuscript. GF: Participated in the design of the study, analysis and interpretations of the findings, drafting the manuscript and write up. All authors reviewed and approved the final manuscript.
